# Prion Protein Is a Key Determinant of Alcohol Sensitivity through the Modulation of N-Methyl-D-Aspartate Receptor (NMDAR) Activity

**DOI:** 10.1371/journal.pone.0034691

**Published:** 2012-04-20

**Authors:** Agnès Petit-Paitel, Baptiste Ménard, Alice Guyon, Vincent Béringue, Jean-Louis Nahon, Nicole Zsürger, Joëlle Chabry

**Affiliations:** 1 Centre National de la Recherche Scientifique, Institut de Pharmacologie Moléculaire et Cellulaire, Valbonne, France; 2 Institut National de la Recherche Agronomique, UR892, Virologie et Immunologie Moléculaires, Jouy-en-Josas, France; INSERM, UMR-S747, France

## Abstract

The prion protein (PrP) is absolutely required for the development of prion diseases; nevertheless, its physiological functions in the central nervous system remain elusive. Using a combination of behavioral, electrophysiological and biochemical approaches in transgenic mouse models, we provide strong evidence for a crucial role of PrP in alcohol sensitivity. Indeed, PrP knock out (PrP^−/−^) mice presented a greater sensitivity to the sedative effects of EtOH compared to wild-type (wt) control mice. Conversely, compared to wt mice, those over-expressing mouse, human or hamster PrP genes presented a relative insensitivity to ethanol-induced sedation. An acute tolerance (i.e. reversion) to ethanol inhibition of N-methyl-D-aspartate (NMDA) receptor-mediated excitatory post-synaptic potentials in hippocampal slices developed slower in PrP^−/−^ mice than in wt mice. We show that PrP is required to induce acute tolerance to ethanol by activating a Src-protein tyrosine kinase-dependent intracellular signaling pathway. In an attempt to decipher the molecular mechanisms underlying PrP-dependent ethanol effect, we looked for changes in lipid raft features in hippocampus of ethanol-treated wt mice compared to PrP^−/−^ mice. Ethanol induced rapid and transient changes of buoyancy of lipid raft-associated proteins in hippocampus of wt but not PrP^−/−^ mice suggesting a possible mechanistic link for PrP-dependent signal transduction. Together, our results reveal a hitherto unknown physiological role of PrP on the regulation of NMDAR activity and highlight its crucial role in synaptic functions.

## Introduction

Alcohol is among the most widely abused drugs in the world. Neuronal mechanisms responsible for the different behavioral responses to ethanol (EtOH) such as tolerance, dependence and intoxication generate intense interest to the scientific community. Alcohol has multiple effects on neurons as it modifies the physiological activity of many receptors and ion channels including γ-aminobutyric acid A (GABA_A_) and N-methyl-D-aspartate (NMDA)[1]. NMDA receptors (NMDARs) are involved in all EtOH-associated phenotypes such as dependence, tolerance or craving indicating that they are pivotal for EtOH-induced behaviors [2]. NMDARs consist of obligatory GluN1 subunits associated with different GluN2 (A–D) subunits. GluN2B is substrate for phosphorylation by members of the Src-protein tyrosine kinases (Src-PTK), mainly fyn. Acute exposure to EtOH inhibits NMDAR activity leading to a decrease in neuronal excitability; however, within minutes after EtOH administration, activated fyn kinase specifically phosphorylates GluN2B subunit in the hippocampus, restoring the channel activity [3], [4]. Consistently, mice lacking the fyn kinase gene are highly sensitive to EtOH sedative effects and do not develop tolerance [5]. Accumulating evidence shows a major role for the NMDARs in EtOH-mediated effects; therefore, all proteins or factors likely to modulate NMDAR functions exhibit an obvious interest in the pathology of alcohol-related disorders. Interestingly, growing data suggest that the prion protein (PrP) could be one of these NMDA modulating factors [6].

Widely expressed in brain, PrP is a membrane-bound, glycophosphatidylinositol (GPI)-anchored protein found primarily in lipid rafts on the cell membrane [7]. Expression of PrP is absolutely required for the neuropathogenesis of a set of fatal neurodegenerative disorders, namely prion diseases [8]. Because of its pivotal role in the etiology of these diseases, considerable efforts have been made to decipher the neurophysiological roles of PrP, which still remain enigmatic [9]. PrP has been involved in a variety of physiological functions ranging from regulation of circadian rythm and sleep [10], [11] to spatial learning [12]. Neurons of PrP-null (PrP^−/−^) mice are highly sensitive to hyper-excitability [13] and excitotoxicity [14], both features closely linked to NMDAR activity. By binding to GluN2D subunit, PrP could prevent NMDAR sustained activity and thus excitotoxicity [6].

Since PrP can stimulate Src-PTK-dependent intracellular signaling pathway [15], [16], which in turn may regulate the NMDAR activity, we hypothesized a possible involvement of PrP in the behavioral sensitivity and acute tolerance to EtOH. We show that PrP^−/−^ mice present exacerbated EtOH sensitivity compared to wild type mice. Conversely, mice over-expressing mouse, human or hamster PrP sequences are less sensitive to the EtOH-induced sedative effects than wild type mice. Electrophysiological and biochemical experiments show that PrP is a key determinant in the establishment of the acute tolerance to EtOH through a fyn-mediated GluN2B-NMDAR activation. In our attempt to decipher the molecular mechanisms underlying this process, we investigated the effects of EtOH on lipid membrane features. We established that EtOH altered lipid raft features in a different way on hippocampal homogenates prepared from EtOH-treated wt and PrP^−/−^ mice, which underlies the implementation of distinct intra-cellular signaling pathways.

**Figure 1:**
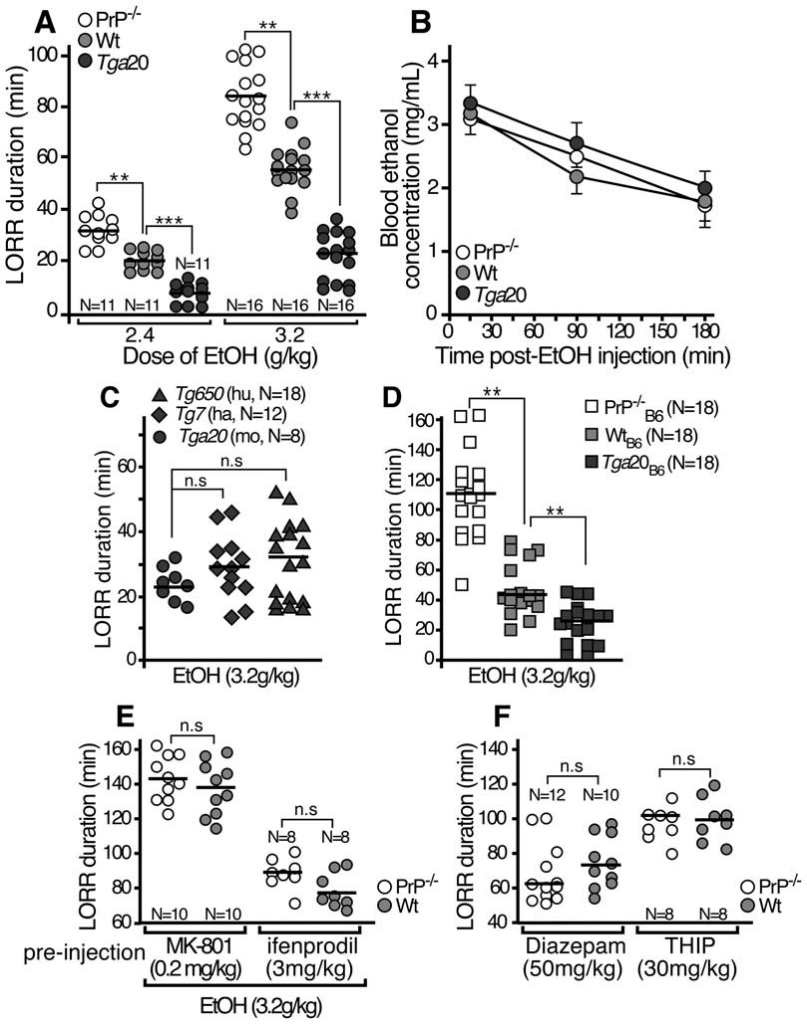
Altered sedative effect of EtOH in PrP^−/−^, PrP-over-expressing mice: relation to NMDAR function. EtOH sensitivity was evaluated by measuring the duration of LORR after *i.p*. injection of 2.4 (**A**, *left*) or 3.2 g/kg EtOH (**A**, *right* and **B**, **C**, **D, E**). In panel **E**, the EtOH injection was preceded by an *i.p*. injection of 0.2 mg/kg of the NMDAR antagonist, MK-801 or 3 mg/kg of ifenprodil. In panel **F**, sedation induced by GABA modulators was evaluated by measuring the duration of LORR after *i.p*. injection of 50 mg/kg diazepam (*left*) or 30 g/kg THIP (*right*). For LORR experiments (panels **A**, **C**, **D**, **E** and **F**), each symbol represents a mouse; the number of mice tested is shown below each condition; the dark line is the median value. The wt mice i.e. C57BL6/J x 129/Sv for panels **A, B, C, E** and **F** or C57BL6/J for panel **D** (noted Wt_B6_) were taken as controls. Significance (p) was calculated using the nonparametric Mann-Whitney test; *p<0.05; **p<0.01; ***p<0.001; n.s; not significant. Blood EtOH concentration (**B**) was performed on four mice of each genotype for each time point and represented as the mean ± SD (*student t* test).

## Materials and Methods

### Animals

In the present study, we used transgenic mouse lines knockout for the PrP gene (PrP^−/−^ [17]), over-expressing (5–8 fold) mouse PrP (*Prnp-a* allele, *tga20* line [18]), human PrP (M129 allele, *tg650* line [19]) or hamster PrP (*tg7* line [20]) on a PrP^−/−^ background. Because PrP^−/−^ mice are on a C57BL6/Jx129/Sv mixed genetic background, we used the hybrid strain of C57Bl6/J x 129/Sv as wt controls. To control for the possible influence of the genetic background, homozygous knockout and over-expressing mouse PrP gene mice onto pure C57BL6/J background were also used [21], [22]. To do so, heterozygote mating pairs, inbred for at least ten generations onto C57BL6/J background, were mated to produce homozygous (noted as PrP^−/−^_B6_ and *tga*20_B6_) and wt (wt_B6_) littermates. All animals were handled in accordance with good animal practice as defined by the relevant national animal welfare bodies, equivalent to the European Convention for the Protection of Vertebrate Animals used for Experimental and other Scientific Purposes (ETS 123). Mouse experimentation protocols were approved by the Nice Sophia Antipolis University regional animal safety committee (CIEPAL-Azur).

**Figure 2:**
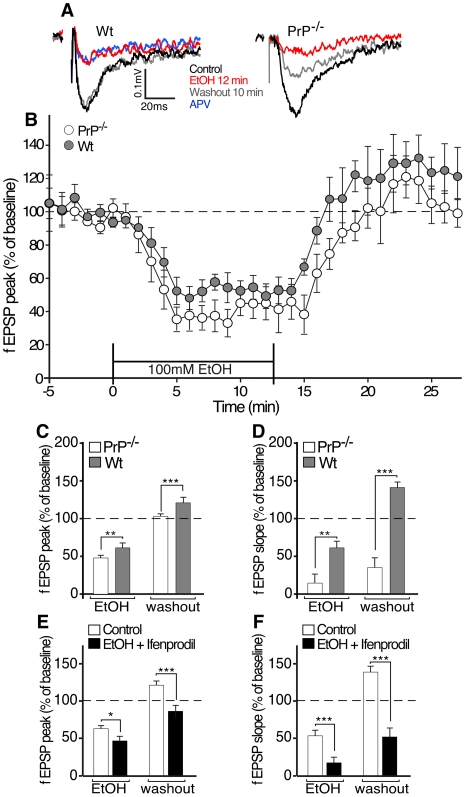
Effect of acute exposure to EtOH on GluN2B-containing NMDARfunction in hippocampal slices of PrP^−/−^ and wt mice. Panel **A** represents typical traces of: NMDAR fEPSP recorded in the presence of CNQX of wt (*left*) and PrP^−/−^ (*right*) hippocampal slices (average of 6 single sweeps) obtained before (baseline, black), during EtOH application for 12 min (red), 10 min after EtOH wash out (grey) or in the presence of the NMDAR antagonist, APV (blue). Time course for normalized maximal peaks (**B**) for fEPSPs in the hippocampus of PrP^−/−^ (open) and wt (grey) mice measured before and during the bath application of 100 mM EtOH. Data are presented as mean ±SEM, percentage of baseline. Curves were significantly different from times 3–25 min using exact and Monte Carlo resampling for the Wilcoxon-Mann-Whitney test (p<0.05). Histograms show the comparison of fEPSPs peaks (**C**) and slopes (**D**) during EtOH application (0–12 min) and after EtOH wash out (17−27 min in **B**). Mean ±SEM, N  =  66, 33 in **C** for wt and PrP^−/−^ respectively. Comparison of fEPSPs peaks (**E**) and slopes (**F**) in wt mouse during 15 min of EtOH application and after wash out with and without 5 µM ifenprodil. Mean ± s.e.m, n = 33, 66 with and without ifenprodil respectively. **p<0.01, ***p<0.001 using a one-way ANOVA followed by a Dunnett's *post hoc* analysis.

### Loss of righting reflex (LORR)

For all behavioral and biochemical experiments, 8- to10-week old male mice were housed in individual cages for a 30 min habituation period then injected *i.p*. with EtOH (2.4 or 3.2 g/kg, 30% or 40% solutions in NaCl 0.9% respectively). After the mice lost the righting reflex, they were put on their backs in their home cage. The duration of LORR was defined as the time from the loss of the righting reflex to the time at which it was regained. Recovery was defined as the time at which mice could right themselves twice in a 30 second period after being placed on their backs [23]. The behavioral room was illuminated with a soft light, and external noise was attenuated. Animals that failed to react to hypnotic effects of EtOH and those presenting a LORR greater than 180 min were excluded from the statistical analysis. Sensitivity to hypnotic effect of 4,5,6,7-tetrahydroisoxazolo[5,4-c]pyridin-3-ol (THIP) and diazepam was assessed using same procedure. Blood samples of ∼50 µl were obtained from tail vein 15, 90 and 180 min after administration of 3.2 g/kg EtOH from independent experiments. Blood EtOH concentration was quantified using the DXC600, Beckmann Coulter.

### Preparation of hippocampus homogenates and western blotting

Subjects were decapitated 5, 20 and 60 min after 3.2 g/Kg EtOH or 5 min after saline (NaCl 0.9%, Ctrl) injection. Hippocampi were dissected and immediately frozen on dry ice. Tissues were homogenized in cold lysis buffer containing 10 mM Tris-HCl pH 7.4, 1% triton X-100, 0.1% CHAPS, 140 mM NaCl, 1 mM EDTA, 1 mM sodium orthovanadate, and protease and phosphatase inhibitor cocktails, then centrifuged at 4000 rpm for 20 min at 4°C. Supernatants (100 µg of proteins) were mixed with the 2X denaturing loading buffer (20 mM Tris-HCl pH 6.8, 10% SDS, 10% glycerol, 10% ß-mercaptoethanol) boiled for 5 min, loaded onto a 10% polyacrylamide gel and then blotted onto a nitrocellulose membrane. Rabbit polyclonal antibodies were used to detect flotilin-1, GluN2B-containing NMDAR and phosphorylated-Tyr1472 GluN2B (p-Y1472) (AbCam, cat. ab41927, ab65875 and ab59205 respectively). Goat monoclonal antibodies to detect fyn were from Santa Cruz (cat. sc-434). Rabbit polyclonal antibodies from Cell Signaling against phospho-Y416Src-PTK cross-react with all Src-PTK members when phosphorylated at the equivalent site (cat. 2101S). Anti-PrP mouse monoclonal antibody, SAF83, was provided by Dr. J. Grassi (CEA, France).

Blots were developed using an enhanced chemoluminescence system with a LAS3000 detector (Fuji). Densitometry analyses were performed with a “*National Institutes of Health*” Image software.

### Isolation of lipid-raft microdomains by sucrose-gradient centrifugation

Hippocampi were lysed for 30 min at 4°C in 0.5 ml of 25 mM Tris-HCl pH 7.5 containing 150 mM NaCl, 1% Triton X-100. Lysates were resuspended in an equal volume of 85% sucrose and placed beneath a discontinuous gradient of sucrose consisting of 4 ml of 35% sucrose and 4 ml of 5% sucrose. Samples were centrifuged at 200 000 g for 16 h at 4°C in a SW 41 rotor, then fractions of 1 ml (9 fractions in total) were collected from the top of the gradient tube (fraction 1) to the bottom (fraction 9). Thirty µl of each fraction were analyzed by SDS-PAGE and western blot.

### Immuno-stainings and confocal laser microscopy on hippocampal neurons in primary culture

Hippocampal neurons from embryonic day 16–17 mice were prepared as described previously [24]. Briefly, dissociated neurons were plated onto glass coverslips pre-coated with polylysine and used after 15–18 days of culture. Neurons were washed three times in PBS and then incubated at 37°C in Earle's buffer (HEPES 25 mM Tris buffered to pH 7.4, NaCl 140 mM, KCl 5 mM, CaCl_2_ 1.8 mM, MgCl_2_ 0.8 mM, glucose 5 mM, BSA 0.01%) in the absence or in the presence of 100 mM EtOH for times indicated in the results. After two washes on ice with ice-cold PBS, neurons were fixed with 4% paraformaldehyde in PBS, 5% sucrose for 30 min at 4°C then washed three times with PBS. After 20 min in PBS, 5% BSA, cells were permeabilized in PBS, 5% BSA containing 0.2% triton X-100. Coverslips were incubated with 1∶200 primary antibodies for 3 h at room temperature in PBS, 1% BSA, followed by incubation with the appropriate fluorescent-conjugated secondary antibody (1∶1000). Coverslips were then mounted on glass slices with Mowiol containing 1 µg/ml Hoescht, a fluorescent specific DNA dye. Each fluorochrome was independently captured with an FV10i scanning confocal microscope (Olympus, France). Images were acquired as single transcellular optical sections and analyzed using Image J software ("National Institutes of Health”).

### Hippocampal slice preparation and electrophysiological recordings

Brains were rapidly collected into ice-cold artificial cerebrospinal fluid (aCSF), containing (in mM): 125 NaCl, 2.5 KCl, 1 MgCl_2_, 2 CaCl_2_, 1.25 NaH_2_PO_4_, 26 NaHCO_3_, pH 7.4, 25 glucose. Sagital slices of hippocampus (300 µM) were cut using a HM650V vibratome (Microm, Germany) and placed in a holding chamber at 34°C for 1 h and bubbled with 95% O_2_, 5% CO_2._ Individual slices were placed in a submerged recording chamber and continuously superfused with “low Mg^2+”^ aCSF (Mg^2+^ concentration lowered to 0.1 mM to unblock the NMDA receptors) at a flow rate of ∼2 ml/min (23°C±1). In response to stimulation of the Schaffer collateral-commissural pathway by a steel bipolar microelectrode, field EPSPs (fEPSPs) from the stratum radiatum of the hippocamal CA1 region were recorded extra cellularly using a glass micropipette filled with aCSF (5–10 MΩ). The stimulus intensity was set to evoke responses which were about half of the magnitude at which a population spike started to appear. Control responses were recorded in the presence of 10 µM 6-cyano-7-nitroquinoxalin 2,3 dione (CNQX, which blocks the non-NMDA component of the fEPSP), for 10 min (one single pulse every 10 s, averaged by groups of 6). Field potentials were recorded using a Axopatch 200B and peaks and slopes amplitudes of fEPSPs were analyzed using pCLAMP software (Axon Instruments). To determine the changes in fEPSP peak and slope amplitudes following 100 mM EtOH, fEPSPs were normalized to control (average peak or slope of 5 min pre-EtOH). Traces were filtered at 1000 Hz with Clampfit software and smoothed by adjacent averaging using Microcal Origin software. CNQX, EtOH and DL-2-aminophosphonovalerate (APV, 10 µM) dissolved in aCSF were applied in the perfusion medium.

### Statistics

Statistical analysis was performed using Statistica software™. For behavior experiments, Mann-Whitney test was used for comparison between groups, each dot representing an animal. For western blot analysis, comparison of mice was performed using the one-way analysis of variance followed by a Dunnett's *post hoc* analysis comparing all data to these obtained with the wt mice in the absence of EtOH taken as control.

## Results

### PrP-null and over-expressing PrP gene mice present altered sedation in response to an acute administration of EtOH

To investigate the involvement of PrP in the sedative effect induced by EtOH, we measured the duration of the loss of righting reflex (LORR) in PrP^−/−^, wt and over-expressing the mouse PrP gene (*tga*20). For both concentrations of EtOH administrated (i.e 2.4 and 3.2 g/kg), the duration of LORR was significantly longer for PrP^−/−^ mice than for wild type (wt) control (Fig. 1A). Conversely, the LORR duration was shorter for *tga*20 mice compared to wt mice (Fig. 1A). Difference between the three mouse strains does not result from differential EtOH metabolism, since no significant difference in the blood EtOH concentration was found over time after EtOH administration (Fig. 1B). To examine the effect of different PrP genes, we also measured the LORR duration in mice over-expressing human and hamster PrP, namely *tg650* and *tg7* respectively (Fig. 1C). Mice over-expressing similar levels (i.e. 5–8 fold over-expression) of mouse, human and hamster PrP presented LORR durations significantly shorter than that of wt mice (Fig. 1A, C). Nevertheless, to control for a possible effect of the genetic background, we performed LORR assay on PrP^−/−^ and *tga20* back-crossed on a pure C57BL6/J genetic background (Fig. 1D). PrP^−/−^_B6_ mice slept much longer and *tga20*_B6_ mice shorter than wt_B6_ mice suggesting that the EtOH-induced phenotype is due to the *Prnp* gene rather than to other genetic factors (Fig. 1D).

In order to examine whether the observed effects of alcohol could involve the NMDA receptors, mice were pretreated with MK-801, a selective NMDA open channel blocker [25], [26] or ifenprodil, a selective blocker of GluN2B. Strikingly, pre-treatment with MK-801 or ifenprodil abolished the difference of LORR between PrP^−/−^ and wt mice suggesting the involvement of NMDAR activity in the PrP-dependent sensibility to EtOH (Fig. 1E). Unlike ifenprodil, pre-treatment with MK-801 markedly potentiated the sedative effect of 3.2 g/kg EtOH both in PrP^−/−^ and wt mice (Fig. 1A, E). In agreement with previous studies, we found that *i.p*. injection of MK-801 (0.2 mg/kg) or ifenprodil (3 mg/kg) never produced sedative effects *per se* ([27], [28] and data not shown).

The GABAergic neurotransmitter system has also been involved in many EtOH's behavioral effects, therefore it was of interest to assess whether sensitivity to GABAergic drugs was altered by the *Prnp* gene deletion. To do so, duration of LORR was measured following administration of diazepam (a non subunit-selective GABA_A_ receptor modulator) and THIP (a selective GABA_A_ receptor agonist) in PrP^−/−^ and wt mice (Fig. 1F). The mouse strains did not differ in their sensitivity to GABA receptor modulators suggesting that *Prnp* gene deletion did not alter significantly the GABA receptor functions. Taken together, our data suggest that the level of PrP expression may control EtOH-induced sedative effects probably through the regulation of the GluN2B-containing NMDAR activity.

### Altered electrophysiological activity of NMDAR in hippocampal slices from PrP^−/−^ mice following EtOH exposure

To ascertain whether exacerbated EtOH sensitivity of PrP^−/−^ mice was due to altered GluN2B activity, we carried out electrophysiological recordings on EtOH-treated hippocampal slices. Exposure of hippocampal slices to EtOH is known to transiently inhibit GluN2B-mediated excitatory post-synaptic potentials (EPSPs) [29]. An acute tolerance caused by the Src-PTK-mediated phosphorylation of GluN2B is rapidly set up, resulting in the potentiation of NMDAR currents [5]. We hypothesized that the difference of LORR phenotype between PrP^−/−^ and wt mice may be due to altered acute tolerance kinetics. To test this hypothesis, we compared the effects of EtOH on NMDA-mediated fEPSPs recorded with extracellular microelectrodes in hippocampal slices from PrP^−/−^ and wt mice (Fig. 2A–D). Bath application of EtOH (100 mM) rapidly depressed NMDAR-mediated fEPSPs in slices from both mice (Fig. 2A–D). The fEPSPs amplitude partially recovered in wt mice after 8 min EtOH indicating the establishment of the acute tolerance, whereas recovery was delayed and lowered in hippocampal slices of PrP^−/−^mice. After EtOH wash out, fEPSP were gradually increased to the baseline for both mice, although the kinetic of recovery was slower for PrP^−/−^ than for wt mice. In average, there was a significant difference between fEPSPs (both peak and slope) from PrP^−/−^ and wt hippocampal slices in EtOH washout (Fig. 2C, D). As previously described [5], ifenprodil eliminated the acute tolerance to EtOH inhibition in hippocampal slices from wt mice (Fig. 2E, F). In summary, the acute tolerance to EtOH inhibition of NMDAR-mediated EPSPs developed more slowly in hippocampus of PrP^−/−^ than in wt mice.

### Altered GluN2B and Src-PTK phosphorylation patterns in hippocampus of PrP^−/−^ mice following EtOH exposure

**Figure 3:**
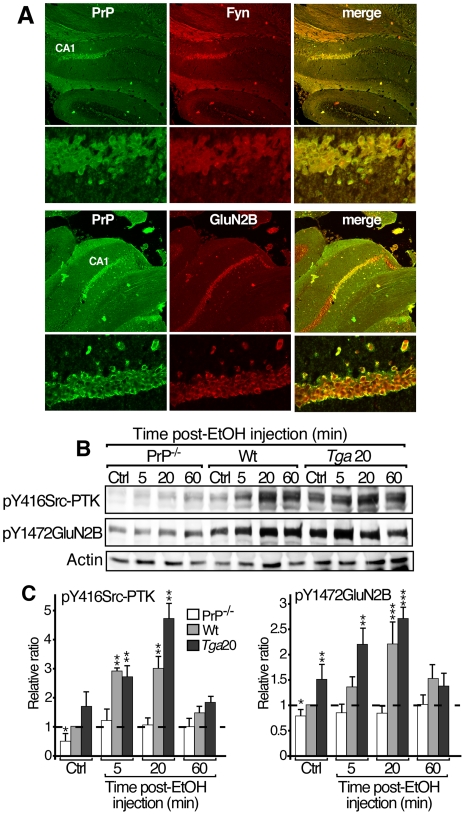
Acute exposure to EtOH transiently increased phosphorylation levels of GluN2B and Src-PTK in hippocampus of PrP-expressing mice but not in that of PrP^−/−^ mice. A. Fixed and permeabilized hippocampus slices were incubated with 1∶200 primary antibodies in PBS 1%BSA containing 0.05% triton X-100 for 3 h, followed by incubation with the appropriate fluorescent-conjugated secondary antibody (1∶1000). Co-labeling with antibodies against PrP (green, *left*) and fyn or GluN2B (red, *middle*) are presented in A. The merge images (*right*) correspond to the superposition of the two fluorescence signals (yellow). B, C. PrP^−/−^, *tga*20 and wt mice were injected *i.p.* with saline and euthanized 5 min later (Ctrl in B and C) or with EtOH (3.2 g/kg) and euthanized 5, 20 or 60 min later (B, C). Solubilized proteins from hippocampus were submitted to SDS-PAGE, blots were then probed with antibodies directed against phospho(p)-Y416Src-PTK, p-Y1472GluN2B, and actin (B). Histograms (C) depict quantification of the levels of phosphorylation of Src-PTK (*left*) and GluN2B (*right*) normalized to actin. Results are representative of 2 animals/condition in 6 independent experiments and presented as mean ± SD. Relative ratio of hippocampus from saline injected wt mouse taken as 1 (N = 6; Dunnett's *post hoc*; *p<0.05; **p<0.01; ***p<0.001).

Since PrP modifies the NMDAR function in hippocampal slices probably *via* a fyn kinase-dependent activation, cell co-localization between these proteins is expected in hippocampal neurons. Employing double immunofluorescence labeling in hippocampal slices, we showed that PrP was strongly co-localized with both GluN2B and fyn in CA1 of mouse hippocampus (Fig. 3A).

The sedative effects of EtOH and the NMDAR activity are closely linked to the phosphorylation of the GluN2B subunit mediated by members of the Src-PTK family [27]. We postulated that the phosphorylation level (i.e. activation) of GluN2B and Src-PTK might be differentially modulated in the hippocampus of PrP^−/−^ and *tga*20 mice as compared to wt after EtOH administration. Mice were *i.p*. injected with saline solution (Ctrl) or 3.2 g/kg of EtOH and euthanized 5, 20 or 60 min later. The phosphorylation levels of GluN2B and Src-PTK rapidly increased in the hippocampus of wt and *tga*20 mice as soon as 5 min post-EtOH injection, and reached a maximal value in 20 min (Fig. 3B, C). In marked contrast, no significant increase was observed in PrP^−/−^ mice (Fig. 3B, C). The anti-phospho-Y416Src-PTK antibody used here does not distinguish between fyn and src; however, the signal is virtually absent in fyn-null mice suggesting that fyn is the predominant kinase in lysates of mouse hippocampus [30]. Interestingly, in basal conditions, both GluN2B and Src-PTK phosphorylation levels were significantly reduced in the hippocampus of PrP^−/−^ and increased in that of *tga*20 as compared to wt mice (Fig. 3B, C, Ctrl) suggesting that PrP is a major regulator of fyn-mediated GluN2B phosphorylation. By quantitative PCR and Western blot experiments, we checked that fyn and GluN2B mRNAs and proteins were indeed expressed at similar levels in hippocampus of the three strains of mice (data not shown).

In summary, we showed that the PrP expression is required to induce fyn-dependent GluN2B phosphorylation in response to acute *in vivo* administration of EtOH.

### Sub-cellular co-localization of PrP, fyn and GluN2B in cultured hippocampal neurons: kinetics of EtOH-induced phosphorylation of Src-PTK and GluN2B

**Figure 4:**
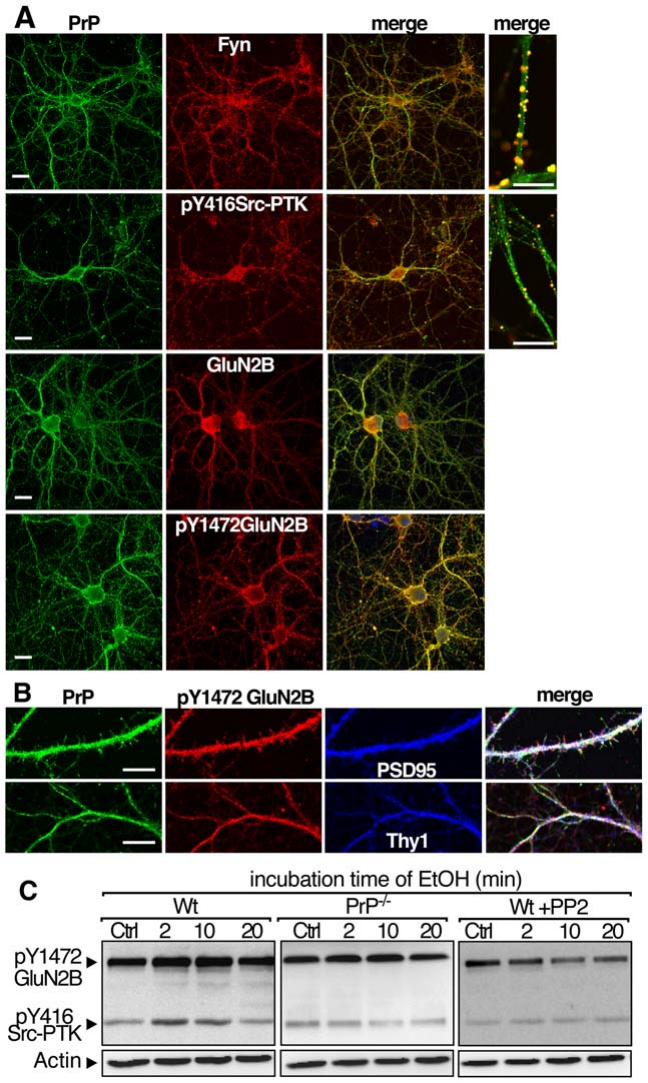
Co-localization of PrP, fyn, GluN2B and their phosphorylated forms in hippocampal neurons in primary culture: Effects of an acute exposure to EtOH on GluN2B phosphorylation level. (A) Hippocampal neurons were co-labeled with antibodies against PrP (green, *left*) and fyn, p-Y416Src-PTK, GluN2B and p-Y1472GluN2B (red, *middle*). The merge images (*right*) correspond to the superposition of the two fluorescence signals (yellow). Nuclei were labeled with the Hoescht reagent and appeared in blue. (B) High magnification of neuronal extensions labeled with antibodies against PrP (green), p-Y1472 GluN2B (red) and PSD95 or Thy1 (blue). The merge images (*right*) correspond to the overlap of the three fluorescence signals (white). *Scale bar* = 10 µm. (C) Hippocampal neurons from wt and PrP^−/−^ mouse embryos were incubated with saline solution (Ctrl) or 100 mM EtOH (in NaCl 0.9%) for the indicated times. Alternatively, PP2 was pre-incubated 15 min before the addition of EtOH. Neurons were washed twice with PBS, lysed and prepared for SDS-PAGE and western blotting analysis. Blots were probed with the antibodies directed against p-Y1472GluN2B, p-Y416Src-PTK and actin to ensure correct protein loading in the different conditions. One representative of four independent experiments is shown.

Immunohistochemistry and confocal microscopy observations were then performed on cultured hippocampal neurons to refine the sub-cellular location of proteins of interest (Fig. 4). PrP co-localized almost perfectly on neuronal extension with both fyn and GluN2B subunit and their phosphorylated isoforms (Fig. 4A). Immunolabelings of PrP and phosphorylated isoform of GluN2B overlapped with PSD95, a specific post-synaptic marker (Fig. 4B). Interestingly, immunolabeling of PrP, overlapped partially with Thy1, a specific marker of lipid rafts (Fig. 4B). Fyn and GluN2B staining were perfectly co-localized with Thy1 (Fig. 4B). Our results indicate that all the proteins of interest are co-localized in lipid rafts of the plasma membrane at a post-synaptic level.

We then investigated whether acute EtOH exposure on cultured hippocampal neurons induced GluN2B phosphorylation in a Src-PTK-dependent manner. In wt hippocampal neurons, phosphorylation levels of both GluN2B and Src-PTK were increased within 2 min of EtOH exposure and returned to pre-stimulation levels within 20 min (Fig. 4C, *left*). When EtOH was co-incubated with PP2, a specific inhibitor of Src-PTK, no change in GluN2B phosphorylation was observed indicating that Src-PTK mediated the EtOH-induced GluN2B phosphorylation (Fig. 4C, *right*). EtOH failed to increase Src-PTK and GluN2B phosphorylation levels in PrP^−/−^ hippocampal neurons confirming that PrP is a major contributor of the EtOH effects in cultured hippocampal neurons (Fig. 4C, *middle*).

### EtOH induced lipid rafts rearrangements on hippocampus of wt but not PrP^−/−^ mice

**Figure 5:**
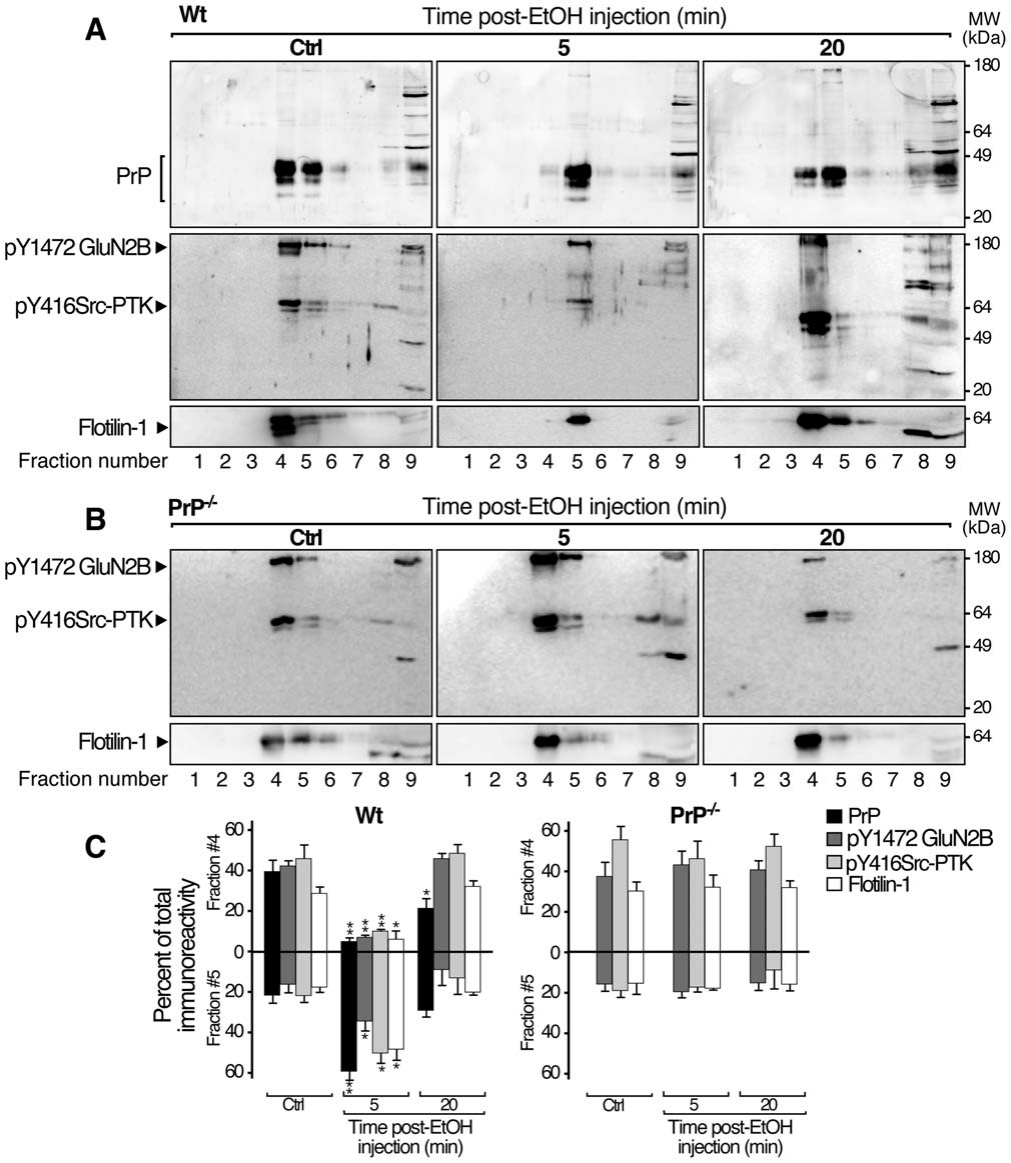
EtOH induced changes in the buoyancy of lipid-raft associated proteins. Lipid-rafts were prepared by sucrose-gradient centrifugation from hippocampus of saline (Ctrl) and EtOH-treated wt (**A**) and PrP^−/−^ (**B**) mice (5, 20 min post-EtOH *i.p* injection). An aliquot of each fraction was analysed by SDS-PAGE and blots were probed with the indicated antibodies. One representative of four independent experiments is shown in panels **A** and **B**. Amounts of immunoreactivity of PrP, p-Y1472GluN2B, p-Y416Src-PTK and flotilin-1 were determined in fractions 4 and 5 and expressed as the percent of total immunoreactivity measured in all fractions for each protein of interest. Results are representative of 2 mice/condition in 4 independent experiments and presented as mean ± SD (N = 4; Dunnett's *post hoc*; EtOH injections versus saline injections: *p<0.05; **p<0.01).

Rearrangement and alterations of lipid membrane have been well documented as early events in EtOH-induced effects [31], [32]. We postulated that EtOH might modify the distribution of proteins of interest differently in hippocampal membranes from PrP^−/−^ and wt mice. To test this hypothesis, lipid raft fractions were prepared by discontinuous sucrose gradient ultracentrifugation from hippocampus of PrP^−/−^ and wt mice in basal conditions and after *i.p*. injection of EtOH, then were analyzed by western blotting. In control conditions, PrP was mainly found in the low-density membrane fraction (Fig. 5A and C, ctrl, fraction 4), together with the phosphorylated forms of GluN2B and Src-PTK and the lipid raft marker, ﬂotilin-1. Similar distribution of these proteins was observed in flotation gradients prepared from PrP^−/−^ mouse hippocampus in control conditions (Fig. 5B and C, ctrl). When flotation gradients were performed on EtOH-treated wt mouse hippocampus, the flotilin-1 labeling shifted toward a lower buoyancy fraction, which might suggest a protein clustering into lipid rafts (Fig. 5A and C 5 min, fraction 5). Five min post-EtOH administration, the immunolabeling of PrP, phosphorylated isoforms of GluN2B and Src-PTK also shifted toward the fraction 5. Twenty min post-EtOH administration, all proteins co-distributed in the fraction 4 as in the control conditions indicating that the EtOH effect on the lipid membrane was transient (Fig. 5A and C, 20 min). Interestingly, no change in buoyancy of proteins of interest was observed after EtOH administration to PrP^−/−^ mice (Fig. 5B), suggesting that PrP could contribute to the formation of such clustering.

## Discussion

**Figure 6:**
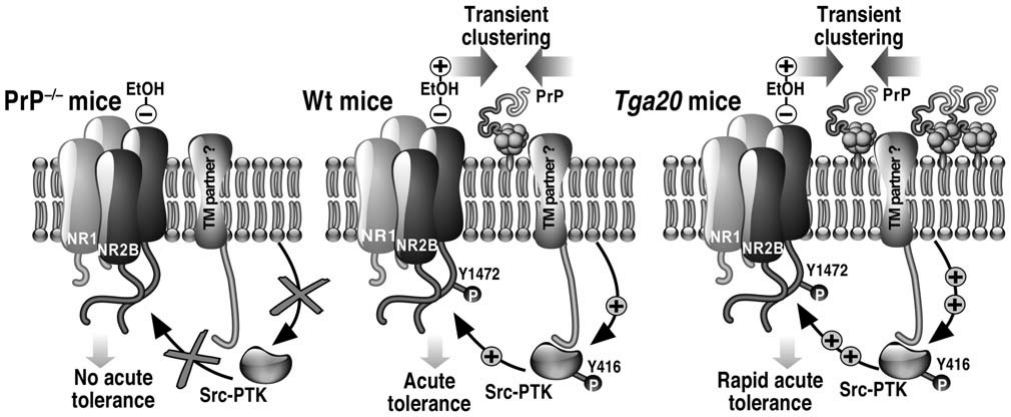
Proposed model of PrP-dependent EtOH effects. In wt mice, acute administration of EtOH inhibits GluN2B-NMDAR currents. An acute tolerance (i.e. reversion) is rapidly set up leading to the repotentiation of NMDAR currents mediated by Src-PTK-dependent phosphorylation of the GluN2B subunit. EtOH provokes the clustering of proteins into lipid rafts allowing interplay of PrP/PrP or PrP/putative trans-membrane partner (TM). Such interplay could be pivotal to activate the Src-PTK (i.e. phosphorylated on tyrosine Y416) that in turn restores NMDAR activity by phosphorylation on tyrosine residue Y1472. In PrP^−/−^ mice, the acute tolerance to EtOH is established slowly because the lack of PrP hinders fyn interplay. Thus, no EtOH-induced activation of the fyn kinase is observed in hippocampus of PrP^−/−^. As a consequence, GluN2B phosphorylation does not occur resulting in the lack of repotentiation of NMDAR currents that may explain, at least partly, the hypersensitivity of PrP^–/−^mice to EtOH. In *tga20* mice, the over-expression of PrP and thus its high concentration into lipid rafts would lead to a constitutive activation of fyn and subsequently to the hyper-phosphorylation of GluN2B-NMDAR.

Expression of PrPc is absolutely required for the neuropathogenesis of a set of dramatic and fatal neurodegenerative disorders, namely TSEs. Because prion-induced neurodegeneration might be a consequence of its loss of function(s), one can assume that neurophysiological function(s) of PrPc must be of prime importance. Despite considerable efforts made in many directions to understand PrPc functions, a clear comprehensive view failed to be achieved. Our results demonstrate that PrP is involved in the neurological effects of acute EtOH exposure by regulating the GluN2B-NMDAR function *via* a fyn-dependent activation pathway, a previously unknown physiological role for this protein. Moreover, our data strongly suggest that PrP plays a pivotal role in the organization of lipid rafts making possible the transduction of intracellular signalings upon acute EtOH administration (Fig. 6).

Based on behavioral experiments, we showed that the EtOH-induced sedative effect is inversely correlated with the level of expression of PrP. Indeed, PrP^−/−^ mice presented a greater sensitivity while *tga20* showed a relative insensitivity to the sedative effect of EtOH as compared to wt mice. The phenotype was manifest in mouse lines on two different genetic backgrounds providing strong evidence that the phenotype is caused by the level of PrP expression *per se*. Like *tga20*, transgenic mice over-expressing human or hamster PrP gene present a relative insensitivity to the EtOH-induced sedative effect as compared to wt mice suggesting that the phenotype is poorly dependent on the PrP sequence.

As expected, in the hippocampus of wt mice, an acute tolerance was rapidly set up through Src-PTK-mediated phosphorylation of the tyrosine residue 1472 of the GluN2B subunit. In PrP^−/−^ mice, EtOH exposure only lead to the inhibition of the channel activity but did not result in Src-PTK and GluN2B-NMDAR phosphorylation. Moreover, acute desensitization or rebond potentiation failed to occur in hippocampal slices from PrP^−/−^ mice after the EtOH wash out. The lack of ethanol-induced intracellular pathway activation may explain the enhanced sensitivity to sedative effects of EtOH in PrP^−/−^ mice. Taken together, we demonstrated that PrP is as key determinant in the EtOH sensitivity through the regulation of GluN2B-NMDAR activity. Deletion and over-expression of PrP profoundly altered behavioral effects of acute EtOH exposure underlining the functional importance of PrP-dependent intracellular signaling cascade. To our knowledge, we provide the first evidence of *in vivo* PrP-mediated intracellular signaling pathway activation.

Proteins, such as PrP, attached to the outer leaflet of the cell membrane *via* GPI anchors are often receptors that mediate cell activation [33]. Because such proteins lack trans-membrane and cytoplasmic domains and therefore cannot directly transduce an intracellular signal, there has been considerable interest in determining how these proteins function. α-PrP antibodies-mediated cross-linking induced fyn activation in a caveolin-dependent pathway [15]. It is possible that EtOH induces the oligomerization of PrP in specific membrane microdomains leading to PrP-dependent transduction. Enriched in potent effectors on both sides of the plasma membrane, lipid rafts are thought as functional platforms favoring signal transduction [34]. An accurate regulation of recruitment into and exclusion outside lipid rafts of signaling proteins is expected for efficient intra-cellular signaling. Owing to its interaction with lipids, EtOH could alter protein recruitment and thus, could provoke subsequently an aberrant downstream response. As an example, EtOH inhibits lipid raft-mediated TCR-signaling in T lymphocytes, resulting in suppression of immune responses [35]? Hence, we demonstrated that EtOH provokes transient lipid rafts rearrangement and thus may trigger specific transduction in neuronal cell. Thus, EtOH may trigger the transient clustering of PrP leading to its oligomerization or alternatively induce interplay with lipid raft components. Remarkably, no buoyancy changes of lipid rafts were observed after acute administration of EtOH to PrP^−/−^ mice suggesting that PrP favors cohesion and interplays of proteins of interest. PrP could be an important factor for the establishment and/or the maintenance of a correct positioning of the different partners, allowing the spatio-temporal sequence of events occurring after an acute EtOH exposure. It is conceivable that the over-expression of PrP (*tga*20 mice) would trigger its constitutive oligomerization at the neuronal surface resulting in sustained fyn activation. As a consequence, GluN2B would be constitutively hyper-phosphorylated in hippocampus of *tga20* mice explaining at least in part the relative insensitivity to EtOH-sedative effects (Fig. 6). Supporting this hypothesis, mice over-expressing fyn present a low sensitivity to EtOH-induced sedation, as *tga20* mice [36].

How PrP and fyn are linked across the membrane remains an intriguing mystery. The neural cell adhesion molecules (N-CAM) could ensure this role. Indeed, the trans-membrane N-CAM isoforms interact both with PrP [37] and fyn [38]. Interestingly, PrP recruits to and stabilizes N-CAM in lipid rafts leading to the activation of fyn [16]. Specific binding sites of EtOH have been recently characterized on the extra-cellular domain of a member of N-CAM family, namely L1 [39]. Further investigations will be required to determine whether N-CAM or other well-known PrP partners such as caveolin or laminin receptor participate to PrP-dependent transduction under EtOH stimulus.

Owing to its localization at the synaptic contacts, PrP could participate to the synaptic physiology [40], [41]. Our study is consistent with such assumption, inasmuch as the deletion of *Prnp* gene results in altered synaptic NMDAR function in response to EtOH exposure. Altogether, our results reinforce the concept of a prominent role of PrP in synaptic function. In addition, the identification of a new partner, namely the PrP, involved in neuronal responses to alcohol should allow the identification of new targets for future drug development for the treatment of alcohol disorders.
